# Discriminatory ability of anthropometric measurements of central fat distribution for prediction of post-prandial hyperglycaemia in patients with normal fasting glucose: the DICAMANO Study

**DOI:** 10.1186/s12967-019-1787-5

**Published:** 2019-02-18

**Authors:** Belén Pérez-Pevida, Jorge M. Núñez-Córdoba, Sonia Romero, Alexander Dimitri Miras, Patricia Ibañez, Neus Vila, María Ángeles Margall, Camilo Silva, Javier Salvador, Gema Frühbeck, Javier Escalada

**Affiliations:** 1 Section of Investigative Medicine, Division of Diabetes, Endocrinology and Metabolism, Imperial College London, 6th Floor, Commonwealth Building, Du Cane Road, London, W12 0NN UK; 2 Department of Endocrinology and Nutrition, Clínica Universidad de Navarra, Pamplona, Spain; 3 Biomedical Research Networking Center for Physiopathology of Obesity and Nutrition (CIBEROBN), ISCIII, Pamplona, Spain; 4 Obesity and Adipobiology Group, Healthcare Research Institute of Navarra (IdiSNA), Pamplona, Spain; 5 Division of Biostatistics, Research Support Service, Central Clinical Trials Unit, Clínica Universidad de Navarra, Pamplona, Spain; 6 Department of Preventive Medicine and Public Health, Medical School, Universidad de Navarra, Pamplona, Spain

**Keywords:** Obesity, Anthropometric measurements, Oral glucose tolerance testing, Insulin resistance, Beta-cell function

## Abstract

**Background and aims:**

Obesity is associated with impaired glucose tolerance which is a risk factor for cardiovascular risk. However, the oral glucose tolerance test (OGTT) is not usually performed in patients with normal fasting glycaemia, thus offering false reassurance to patients with overweight or obesity who may have post-prandial hyperglycaemia. As an alternative to resource demanding OGTTs, we aimed to examine the predictive value of anthropometric measures of total and central fat distribution for post-prandial hyperglycaemia in patients with overweight and obesity with normal fasting glycaemia enrolled in the DICAMANO study.

**Methods:**

We studied 447 subjects with overweight/obesity with a fasting glucose value ≤ 5.5 mmol l^−1^ (99 mg dl^−1^) and BMI ≥ 25 kg/m^2^ who underwent a 75-g OGTT. Post-prandial hyperglycaemia was defined as a glucose level ≥ 7.8 mmol l^−1^ (140 mg dl^−1^) 2-h after the OGTT. The anthropometric measurements included body mass index, body adiposity index, waist circumference, neck circumference, waist-to-hip ratio and waist-to-height ratio.

**Results:**

The prevalence of post-prandial hyperglycaemia was 26%. Mean 1-h OGTT glucose levels, insulin resistance and beta cell dysfunction was higher in those subjects in the highest tertile for each anthropometric measurement, irrespective of fasting glucose level. Central fat depot anthropometric measurements were strongly and independently associated with an increased risk of post-prandial hyperglycaemia. After multivariable-adjustment for fasting plasma glucose level, smoking, and physical activity level, the odds ratio (95% confidence intervals) for the presence of post-prandial hyperglycaemia for neck circumference, waist circumference and waist-to-height ratio were 3.3 (1.4, 7.7), 2.4 (1.4, 4.4) and 2.5 (1.4, 4.5), respectively.

**Conclusions:**

In this large and comprehensively phenotyped cohort, one in four subjects had post-prandial hyperglycaemia despite normal fasting glycaemia. Anthropometric indices of central fat distribution were strongly and independently associated with an increased risk of post-prandial hyperglycaemia. These results support the association between central adiposity and glucose derangements and demonstrate the clinical usefulness of anthropometric measurements as screening tools for the selection of patients who are most likely to benefit from an OGTT.

*Trial registration* ClinicalTrials.gov Identifier: NCT03506581. Registered 24 April 2018—Retrospectively registered, https://clinicaltrials.gov/ct2/show/NCT03506581

**Electronic supplementary material:**

The online version of this article (10.1186/s12967-019-1787-5) contains supplementary material, which is available to authorized users.

## Background

Evidence from well-conducted observational studies supports testing for prediabetes and risk for future type 2 diabetes mellitus (T2DM) in asymptomatic adults with overweight or obesity and who have one or more additional risk factors for T2DM [1]. In addition to glycated haemoglobin, the 2-h oral glucose tolerance test (OGTT) represents the basis for screening and/or diagnosis of post-prandial hyperglycaemia.

Impaired glucose tolerance is a risk factor for cardiovascular disease [2]. However, despite the fact that 40% of subjects who develop T2DM have normal fasting glucose at baseline [2], there is still controversy as to whether these patients should undergo an OGTT. Moreover, due to resource demands, the OGTT is not routinely performed in patients with normal fasting glucose, thus potentially missing patients with post-prandial impaired glucose tolerance or frank T2DM. This raises the unmet need for readily available and inexpensive markers that can be used to screen patients that are most likely to have post-prandial hyperglycaemia and therefore benefit the most from an OGTT. These patients could benefit from lifestyle interventions designed to reduce insulin resistance and preserve β-cell function in an attempt to prevent T2DM.

Anthropometric measurements, such as body mass index (BMI), body adiposity index, neck circumference, waist circumference, waist-hip-ratio or waist-to-height ratio, are simple, reproducible and inexpensive tools that can be used as surrogates of central adiposity. To the best of our knowledge, no previous study has determined their predictive value for post-prandial hyperglycaemia (impaired glucose tolerance and T2DM) in patients with overweight or obesity with normal fasting glycaemia [1].

Therefore, in the present study we comprehensively phenotyped a large cohort of patients with overweight and obesity enrolled in the “Discovering Carbohydrate Metabolism Alterations in Normoglycemic Obese patients study” (DICAMANO); clinicaltrials.gov identifier NCT03506581) in order to assess the prevalence of post-prandial hyperglycaemia in individuals with normal fasting glycaemia and examine the predictive value of anthropometric measurements as screening tools for the selection of patients who are most likely to benefit from an OGTT.

## Materials and methods

### Study design and participants

**Fig. 1:**
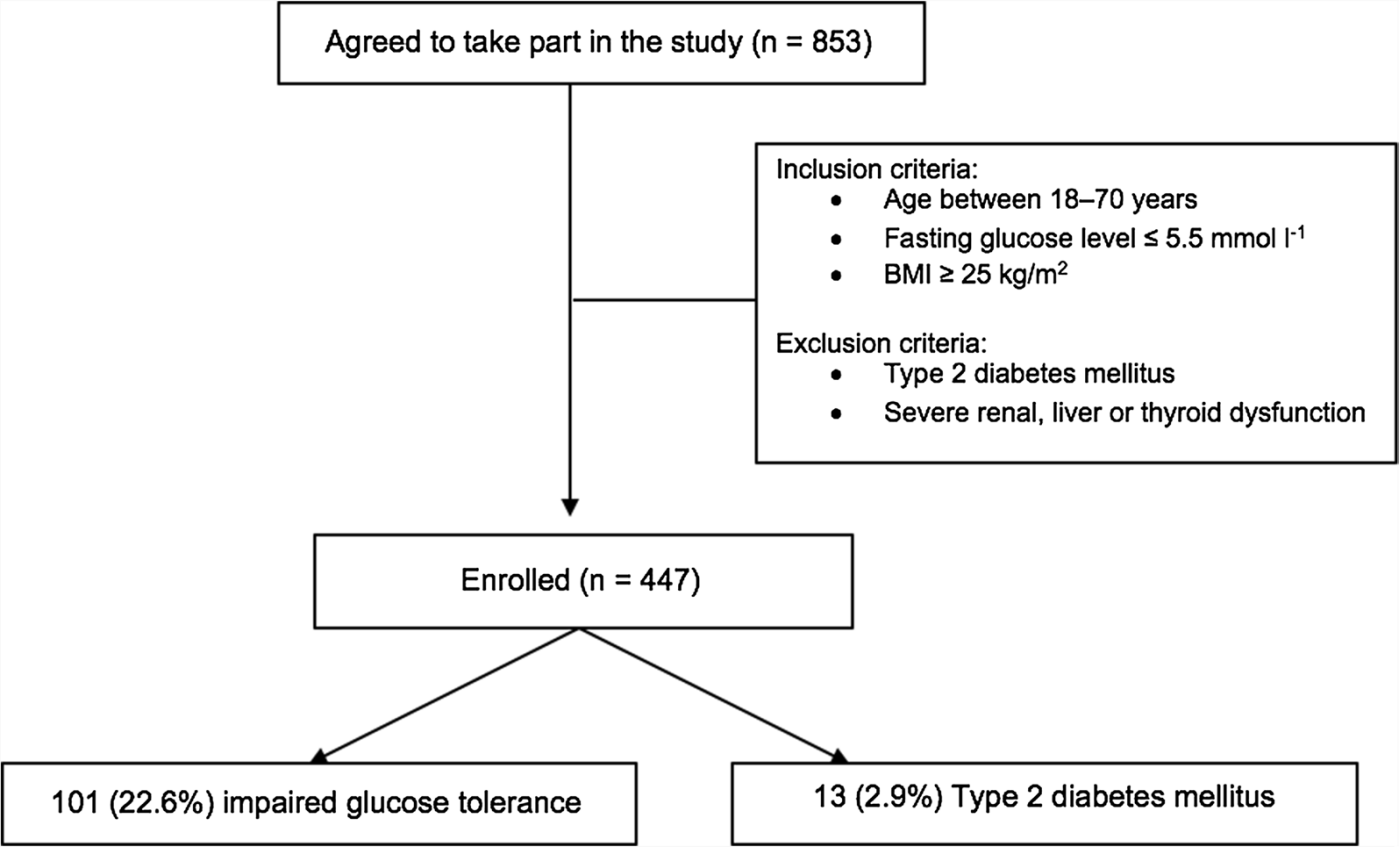
Recruitment and inclusion criteria of patients in the study

Subjects aged 18–70 years, who attended the Department of Endocrinology and Nutrition of the Clínica Universidad de Navarra from 2009 to 2014 for a check-up were offered to participate in the DICAMANO study; 853 subjects agreed to take part. Only those individuals with a normal fasting glucose level (≤ 5.5 mmol l^−1^) and BMI ≥ 25 kg/m^2^ were analysed. Subjects with previous T2DM or severe renal, liver or thyroid dysfunction were excluded (Fig. 1). Participants were instructed to temporarily discontinue any medication known to affect glucose or lipid metabolism for 48 h. On the day of the study visit, each subject had a complete routine clinical assessment to evaluate the presence of cardiovascular, respiratory, renal or endocrine disorders. All patients underwent a 75-g OGTT with a concomitant anthropometric study, blood pressure monitoring and lipid profile analyses. They were classified by glucose tolerance on the basis of blood glucose levels according to ADA diagnostic criteria for T2DM (2017) [1]. Post-prandial hyperglycaemia was defined as a 2-h OGTT glucose level ≥ 7.8 mmol l^−1^ (140 mg dl^−1^). The experimental design was approved, from an ethical and scientific standpoint, by the Research Ethics Committee of the University of Navarra Clinic. Written informed consent was obtained from all participants.

### Anthropometric measurements and physical activity

Anthropometric measurements as well as venesection were performed on the same day. All measurements were performed with participants dressed in light clothing and barefoot. Height was measured to the nearest 0.1 cm with a Holtain stadiometer (Holtain Ltd., Crymych, UK), while body weight was measured with a calibrated electronic scale to the nearest 0.1 kg. Waist circumference was measured at the midpoint between the iliac crest and the rib cage on the mid-axillary line, and hip circumference at the level of the greater trochanters was measured to the nearest millimetre using a flexible tape. Total body fat was evaluated by BMI (kg/m^2^) and body adiposity index ([hip circumference/height^1.5^] − 18), which has been shown to correlate very well with body fat percentage measured by dual-energy X-ray absorptiometry [3]. Body fat distribution was evaluated by waist-to-hip ratio (waist circumference divided by hip circumference) waist-to-height ratio (waist circumference divided by height) and neck circumference. In addition, the physical activity level (PAL) was estimated by a validated questionnaire taking into account physical activity at home, at work and daily leisure time [4].

### Laboratory measurements

Blood samples were collected after 10–12 h overnight fast. Serum glucose was analysed by an automated analyser (Roche/Hitachi Modular P800), with quantification being based on the enzymatic colorimetric reactions described by Trinder [5]. Insulin was measured by means of an enzyme-amplified chemiluminescence assay (IMMULITE^®^, Diagnostic Products Corp., Los Angeles, CA, USA).

### Assessment of insulin sensitivity and ß-cell function

All participants underwent a 2-h OGTT with an oral bolus containing 75 g of anhydrous glucose. Blood samples were obtained at 0, 30, 60, 90, and 120 min for the measurement of glucose and insulin concentrations. Basal insulin resistance was calculated by the homeostasis model assessment of insulin resistance index (HOMA-IR = fasting insulin concentration (I0) * fasting glucose concentration (G0))/405) and the Quantitative Insulin Sensitivity Check Index (QUICKI = 1/[(log(I0) + log(G0)]) [6]. Whole-body insulin sensitivity was estimated by the Matsuda index (insulin sensitivity index (ISI) as 10,000/sq rt of [G0 * I0] * [mean glucose concentration * mean insulin concentration during the OGTT]) [6]. ß-cell function was estimated by the Disposition Index (DI), a measure of insulin secretion during the prevailing level of insulin action relative to the degree of insulin resistance (ΔI0 − 30/ΔG0 − 30 * 1/I0) [6].

### Statistical analysis

For each anthropometric measurement, patients were categorized according to tertiles, with the lowest, second and top tertiles labelled low, intermediate, and high categories, respectively. BMI that was categorized into three predefined tertiles (< 30 kg/m^2^, ≥ 30 kg/m^2^ to < 35 kg/m^2^, and ≥ 35 kg/m^2^). Descriptive statistics were computed for all variables based on the categories mentioned above. Mean 2-h OGTT glucose levels by anthropometric tertiles were compared using analysis of covariance (ANCOVA) to adjust for fasting glucose concentrations.

We used logistic regression models to evaluate the association between the anthropometric measurements and the risk of post-prandial hyperglycaemia. The lowest tertile of the anthropometric measurements served as the reference category. The odds ratios (OR) and their corresponding 95% confidence intervals (95% CI) were additionally adjusted for age, gender, fasting plasma glucose level, and traditional cardiometabolic risk factors, including smoking and physical activity level. Statistical evaluation of the potential modification effect of gender was evaluated.

## Results

### Clinical and metabolic characteristics of the cohort

Mean age was 41 years and 65% of the patients were women. All 447 patients had overweight or obesity with a median BMI of 33 kg/m^2^. Despite the normoglycemic baseline status of the 447 patients examined, 114 (25.5%) had post-prandial hyperglycaemia, 101 (22.6%) had impaired glucose tolerance and 13 (2.9%) T2DM. Demographic and clinical characteristics of the study population by tertiles of anthropometric measurements are presented in Table 1. Patients with higher anthropometric measurements were significantly more likely to be men.

**Table 1:** Demographic and clinical characteristics by categories of anthropometric measurements

	Body mass index (kg/m^2^)				Waist circumference (cm)				Waist-to-hip ratio			
	Low (25.1–29.9)	Intermediate (30.0–34.9)	High (> 35)	p-value	Low (73–100)	Intermediate (101–114)	High (115–170)	p-value	Low (0.71–0.88)	Intermediate (0.89–0.97)	High (0.98–1.30)	p-value
N	120	155	172		156	149	142		150	156	141	
Age, years	40 (14)	42 (14)	40 (13)	0.229	38 (13)	42 (14)	43 (14)	*0.005*	35 (12)	41 (13)	47 (13)	*<* *0.001*
Gender, women, n (%)	91 (76)	100 (64)	100 (58)	*0.008*	132 (85)	101 (68)	58 (41)	*<* *0.001*	142 (95)	108 (69)	41 (29)	*<* *0.001*
Smoking, n (%)	29 (24)	40 (26)	36 (21)	0.571	41 (26)	29 (19)	35 (25)	0.345	31 (21)	42 (27)	32 (23)	0.419
Physical activity level, n (%)												
Sedentary or light activity	84 (70)	118 (76)	140 (81)	*0.050*	115 (74)	109 (73)	118 (83)	0.133	114 (76)	112 (72)	116 (82)	0.229
Active or moderately active	34 (28)	37 (24)	29 (17)		39 (25)	39 (26)	22 (15)		35 (23)	41 (26)	24 (17)	
Vigorous or vigorously active	0 (0)	0 (0)	0 (0)		0 (0)	0 (0)	0 (0)		0 (0)	0 (0)	0 (0)	
Unknown	2 (2)	0 (0)	3 (2)		2 (1)	1 (1)	2 (1)		1 (1)	3 (2)	2 (1)	
Fasting plasma glucose level, mmol l^−1^	5.1 (0.4)	5.0 (0.3)	5.1 (0.3)	0.862	5.0 (0.4)	5.0 (0.3)	5.1 (0.3)	0.408	4.9 (0.4)	5.0 (0.3)	5.1 (0.3)	*<* *0.001*
Basal insulin, pmol l^−1^	56.9 (55.5)	76.4 (76.4)	97.2 (62.5)	*<* *0.001*	62.5 (62.5)	76.4 (69.4)	104.2 (62.5)	*<* *0.001*	69.4 (76.4)	76.4 (55.5)	97.2 (62.5)	*<* *0.001*
HOMA-IR	1.8 (1.6)	2.4 (2.6)	3.2 (2)	*<* *0.001*	1.9 (2)	2.5 (2.3)	3.4 (2.1)	*<* *0.001*	2.2 (2.7)	2.3 (1.7)	3.2 (2)	*<* *0.001*
Insulin sensitivity index	5.8 (3.6)	4.9 (3)	3.6 (2.8)	*<* *0.001*	5.9 (3.6)	4.5 (2.8)	3.3 (2.6)	*<* *0.001*	5.8 (3.6)	4.8 (3.2)	3.2 (2)	*<* *0.001*
Disposition index	6 (5.1)	5.2 (4.6)	4.3 (5.4)	*<* *0.022*	6 (5)	5.2 (6.2)	3.9 (3.3)	*0.001*	6.3 (6.6)	5 (4.4)	3.8 (3.1)	*<* *0.001*
Quantitative insulin sensitivity check index	0.36 (0.04)	0.35 (0.04)	0.33 (0.04)	*0.001*	0.37 (0.04)	0.35 (0.04)	0.33 (0.04)	*<* *0.001*	0.36 (0.05)	0.35 (0.04)	0.33 (0.04)	*<* *0.001*
Insulinogenic index	1.2 (1.6)	1.2 (0.9)	1.5 (1.6)	0.092	1.2 (1.4)	1.3 (1.5)	1.5 (1.3)	0.258	1.2 (1.7)	1.3 (1.4)	1.4 (1)	0.712

	Waist-to-height ratio				Neck circumference (cm)			
	Low (0.47–0.61)	Intermediate (0.62–0.68)	High (0.69–1.11)	p-value	Low (30–35)	Intermediate (36–40)	High (41–57)	p-value
N	167	145	135		153	156	138	
Age, years	37 (12)	42 (13)	44 (14)	*<* *0.001*	39 (13)	40 (14)	44 (13)	*0.004*
Gender, women, n (%)	120 (72)	93 (64)	78 (58)	*0.037*	153 (100)	121 (77)	17 (12)	*<* *0.001*
Smoking, n (%)	39 (23)	36 (25)	30 (22)	0.875	38 (25)	31 (20)	36 (26)	0.405
Physical activity level, n (%)								
Sedentary or light activity	117 (70)	110 (76)	115 (85)	*0.013*	100 (72)	118 (75)	114 (76)	0.154
Active or moderately active	48 (29)	34 (23)	18 (13)		42 (27)	35 (22)	23 (17)	
Vigorous or vigorously active	0 (0)	0 (0)	0 (0)		0 (0)	0 (0)	0 (0)	
Unknown	2 (1)	1 (1)	2 (1)		1 (1)	3 (2)	1 (1)	
Fasting plasma glucose level, mmol l^−1^	5.1 (0.3)	4.9 (0.3)	5.1 (0.3)	*0.015*	4.9 (0.4)	5.0 (0.3)	4.6 (0.3)	*0.004*
Basal insulin, pmol l^−1^	62.5 (62.5)	83.3 (69.4)	104.2 (62.5)	*<* *0.001*	62.5 (76.4)	69.4 (55.5)	104.2 (55.5)	*<* *0.001*
HOMA-IR	1.9 (2.1)	2.6 (2.2)	3.3 (2.2)	*<* *0.001*	2.1 (2.5)	2.3 (1.9)	3.4 (1.9)	*<* *0.001*
Insulin sensitivity index	5.9 (3.6)	4.3 (2.6)	3.5 (2.8)	*<* *0.001*	6 (3.6)	4.8 (3.1)	3 (1.8)	*<* *0.001*
Disposition index	6.1 (5.3)	5.1 (5.9)	3.7 (3.3)	*<* *0.001*	6.6 (6)	4.7 (5.2)	3.8 (3.1)	*<* *0.001*
Quantitative insulin sensitivity check index	037 (0.04)	0.35 (0.04)	0.34 (0.04)	*<* *0.001*	0.37 (0.05)	0.35 (0.04)	0.33 (0.03)	*<* *0.001*
Insulinogenic index	1.2 (1.3)	1.4 (1.6)	1.4 (1.3)	0.366	1.3 (1.5)	1.2 (1.3)	1.5 (1.3)	0.134

Data presented as mean ± SD

Italic values refers to statistically significant difference as compared with the lowest tertile (reference category)

### Insulin sensitivity and ß-cell function

**Fig. 2:**
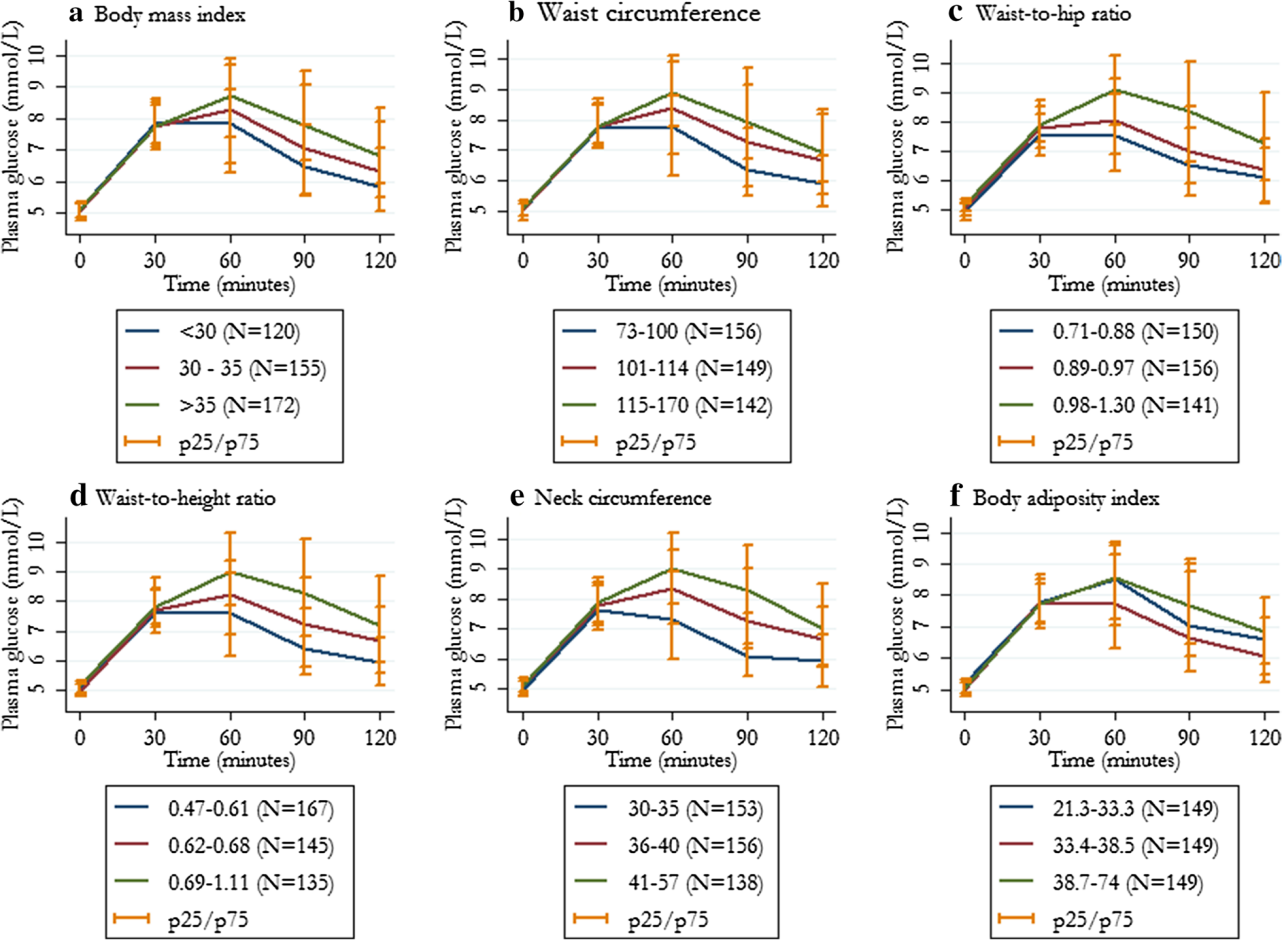
Variation of plasma glucose (mmol l^−1^) during a 2-h 75-g oral glucose tolerance test stratified by tertiles of anthropometric measurements

Fasting plasma glucose levels ranged from 3.7 to 5.5 mmol l^−1^ (66–99 mg dl^−1^), and were similar across tertiles of anthropometric measurements. Higher levels of anthropometric measurements were associated with significantly higher HOMA-IR levels, lower insulin sensitivity index, and lower ß-cell function (Table 1). Figure 2 shows the variation of plasma glucose during a 2-h OGTT stratified by tertiles of anthropometric measurements. Mean 2-h OGTT glucose levels gradually increased across categories of each anthropometric measurement, irrespective of fasting glucose level (Fig. 2; Additional file 1: Table S1). One-hour post-prandial glucose levels were significantly higher at the higher anthropometric measurements tertiles (Additional file 1: Table S1). Those subjects with a 60-min glucose ≥ 8.6 mmol l^−1^ (mg dl^−1^) had the worst lipid profile, greater rates of hypertension and non-alcoholic fatty liver disease, higher insulin resistance and ß-cell dysfunction as compared to subject with normal 1-h value (Table 2).

**Table 2:** Metabolic profile according to 1-h post-prandial glucose levels

	1-h glucose levels after OGTT		
	≤ 8.6 mmol l^−1^	> 8.6 mmol l^−1^	p-value
N (%)	256 (57.3)	191 (42.7)	*0.022*
Hypertension N (%)	25 (10)	49 (26)	*< 0.001*
Obstructive Sleep Apnea Syndrome, N (%)	42 (16)	66 (35)	*< 0.001*
Non-alcoholic fatty liver disease, N (%)	50 (19)	67 (35)	*0.001*
HOMA-IR	2.3 (2.2)	2.9 (2.2)	*0.011*
Insulin sensitivity index	5.4 (3.5)	3.6 (2.5)	*0.005*
Disposition index	6.9 (5.9)	2.6 (1.6)	*< 0.001*
Quantitative insulin sensitivity check index	0.36 (0.04)	0.34 (0.04)	*0.005*
Insulinogenic index	1.6 (1.7)	0.9 (0.7)	*< 0.001*
Total cholesterol, mmol l^−1^	4.9 (0.9)	5.2 (0.9)	*0.022*
LDL cholesterol, mmol l^−1^	2.9 (0.8)	3.2 (0.8)	*0.033*
HDL cholesterol, mmol l^−1^	1.5 (0.4)	1.3 (0.3)	*0.016*
Triglycerides, mmol l^−1^	1.1 (0.6)	1.4 (0.7)	*< 0.001*
Triglycerides/HDL cholesterol ratio	2 (1.6)	2.6 (1.9)	*< 0.001*

Data presented as mean ± SD

Italic values refers to statistically significant difference between groups

### Correlations between anthropometric measurements and post-prandial hyperglycaemia

The highest anthropometric measurements were associated with an increased risk of post-prandial hyperglycaemia after multivariable adjustment. The highest correlation with post-prandial hyperglycaemia was found among those anthropometric measurements assessing fat distribution (waist-to-hip ratio, waist-to-height ratio and neck and waist circumferences) as compared with those surrogates of total body fat (BMI and body adiposity index) (Table 3).

**Table 3:** Associations between anthropometric measurements and risk of post-prandial hyperglycaemia in normoglycemic patients

Anthropometric measurement	Category			P for trend
	Low	Intermediate	High	
Total body fat				
Body mass index, median (minimum, maximum)	27.7 (25.1, 29.9)	32.5 (30.0, 34.8)	39.7 (35.1, 80.1)	
N	120	155	172	
Cases of post-prandial hyperglycaemia (%)	17.5	25.8	30.8	
OR (95% CI)	1.0 (reference)	1.6 (0.9–3.0)	2.1 (1.2–3.7)	*0.014*
Age- and gender-adjusted OR (95% CI)	1.0 (reference)	1.4 (0.8–2.6)	2.0 (1.1–3.5)	*0.023*
Multivariable-adjusted OR (95% CI)^a^	1.0 (reference)	1.4 (0.7–2.5)	2.0 (1.1–3.6)	*0.018*
Body adiposity index, median (minimum, maximum)	29.8 (21.3, 33.3)	36.1 (33.4, 38.5)	42.8 (38.7, 74.0)	
N	149	149	149	
Cases of post-prandial hyperglycaemia (%)	28.9	20.1	27.5	
OR (95% CI)	1.0 (reference)	0.6 (0.4–1.1)	0.9 (0.6–1.6)	0.816
Age- and gender-adjusted OR (95% CI)	1.0 (reference)	0.9 (0.5–1.7)	1.5 (0.8–2.9)	0.155
Multivariable-adjusted OR (95% CI)^a^	1.0 (reference)	0.9 (0.5–1.6)	1.5 (0.8–2.8)	0.95
Central fat distribution				
Neck circumference, median (minimum, maximum)	34 (30, 35)	38 (36, 40)	44 (41, 57)	
N	153	156	138	
Cases of post-prandial hyperglycaemia (%)	15.7	24.4	37.7	
OR (95% CI)	1.0 (reference)	1.7 (0.9–3.1)	3.3 (1.9–5.7)	*<* *0.001*
Age- and gender-adjusted OR (95% CI)	1.0 (reference)	1.7 (0.9–3.1)	3.3 (1.4–7.7)	*0.005*
Multivariable-adjusted OR (95% CI)^a^	1.0 (reference)	1.8 (1.0–3.3)	3.3 (1.4–7.7)	*<* *0.001*
Waist circumference, median (minimum, maximum)	93 (73, 100)	107 (101, 114)	124 (115, 170)	
N	156	149	142	
Cases of post-prandial hyperglycaemia (%)	14.7	28.2	34.5	
OR (95% CI)	1.0 (reference)	2.3 (1.3–4.0)	3.1 (1.7–5.3)	*<* *0.001*
Age- and gender-adjusted OR (95% CI)	1.0 (reference)	1.9 (1.1–3.5)	2.3 (1.3–4.3)	*0.010*
Multivariable-adjusted OR (95% CI)^a^	1.0 (reference)	2.0 (1.1–3.6)	2.4 (1.3–4.4)	*0.010*
Waist-to-hip ratio, median (minimum, maximum)	0.83 (0.71, 0.88)	0.93 (0.89, 0.97)	1.04 (0.98, 1.30)	
N	150	156	141	
Cases of post-prandial hyperglycaemia (%)	17.3	18.6	41.8	
OR (95% CI)	1.0 (reference)	1.1 (0.6–2.0)	3.4 (2.0–5.9)	*<* *0.001*
Age- and gender-adjusted OR (95% CI)	1.0 (reference)	0.9 (0.5–1.7)	2.4 (1.2–4.7)	*0.010*
Multivariable-adjusted OR (95% CI)^a^	1.0 (reference)	0.9 (0.5–1.7)	2.3 (1.2–4.7)	*0.011*
Waist-to-height ratio, median (minimum, maximum)	0.57 (0.47, 0.61)	0.65 (0.62, 0.68)	0.76 (0.69, 1.11)	
N	167	145	135	
Cases of post-prandial hyperglycaemia (%)	15.6	26.2	37.0	
OR (95% CI)	1.0 (reference)	1.9 (1.1–3.4)	3.2 (1.9–5.5)	*<* *0.001*
Age- and gender-adjusted OR (95% CI)	1.0 (reference)	1.6 (0.9–2.9)	2.5 (1.4–4.4)	*0.001*
Multivariable-adjusted OR (95% CI)^a^	1.0 (reference)	1.6 (0.9–2.9)	2.5 (1.4–4.5)	*0.001*

Italic values refers to statistically significant difference as compared with the lowest tertile (reference category)

*CI* confidence interval, *OGTT* oral glucose tolerance test

^a^Additionally, adjusted for fasting plasma glucose level, smoking, and physical activity

Compared with the lowest category of BMI, the age and gender-adjusted OR (95% CI) were 1.4 (0.8; 2.6) and 2.0 (1.1; 3.5) for the intermediate and high category of BMI, respectively (Table 3). Further adjustments for fasting plasma glucose level, smoking, physical activity and gender did not significantly alter these ORs. With regards to the body adiposity index, despite the fact that the highest category showed greater risk of post-prandial hyperglycaemia, this association was not statistically significant (Table 3).

Neck circumference was significantly associated with an increased risk of post-prandial hyperglycaemia with an unadjusted OR (95% CI) of 3.3 (1.9; 5.7) for the highest category, compared with the lowest one. Further adjustments for age, gender, fasting glucose level, smoking, and physical activity did not alter the significance of this association. (Table 3) The multivariable-adjusted OR (95% CI) for the highest categories of neck circumference was significantly higher in men compared to women at 4.0 (1.3; 12.5) and 1.4 (0.4; 4.9), respectively.

Waist circumference, waist-to-hip ratio and waist-to-height ratio were also significantly associated with the risk of post-prandial hyperglycaemia, although with lowest OR compared to neck circumference. The multivariable-adjusted OR (95% CI) for the highest categories of waist circumference, waist-to-hip ratio and waist-to-height ratio were 2.4 (1.3; 4.4), 2.3 (1.2; 4.7), 2.5 (1.4, 4.5), respectively (Table 3). No evidence for an interaction effect between them and gender was observed.

## Discussion

### Key findings

In this study, we comprehensively phenotyped a large cohort of patients with overweight and obesity and found that despite having normal fasting glycaemia the prevalence of post-prandial glucose intolerance or frank T2DM was surprisingly high at 26%. We also demonstrated that readily performed anthropometric measurements including neck circumference, waist-to-hip ratio and waist-to-height ratio were strongly correlated with post-prandial hyperglycaemia independently of fasting glucose level, smoking and physical activity level. Such measurements could therefore be used in standard clinical care to select patients with overweight or obesity that are most likely to benefit from an OGTT. Indeed, the combination of 1-h post-prandial glucose and anthropometric measurements could be used as an even better predictive marker of impaired glucose tolerance and T2DM compared to the full 2-h OGTT.

### Central fat depot and associated metabolic derangement

BMI is the most frequently used tool for the diagnosis and classification of obesity, but it is only a surrogate measure of body adiposity and does not provide an accurate measure of body composition [7–10]. Body adiposity index is another measure of total adiposity which has been shown to correlate strongly with body fat percentage measured by dual-energy X-ray absorptiometry [3]. Even though BMI and body adiposity index were associated with post-prandial hyperglycaemia, anthropometric measurements of central fat distribution were better predictors. These results underscore the importance of central obesity in the development of abnormal glucose metabolism and T2DM for adults with normal fasting glycaemia. Our findings are consistent with previously reported associations between body fat and prediabetes as well as the predictive value of abdominal fat distribution for T2DM and cardiovascular risk beyond that explained by traditional cardiometabolic risk factors) [11–13].

### Anthropometric measurements: predictors of post-prandial hyperglycaemia

In our study neck circumference, waist circumference and waist-to-height ratio showed the strongest correlation with post-prandial hyperglycaemia independently of fasting glucose level, smoking and physical activity level. Out of all anthropometric measurements, neck circumference correlated most strongly with post-prandial hyperglycaemia in both genders but even more so in men whereas this gender interaction was not observed in the other anthropometric measurements. Neck circumference is a surrogate of upper-body subcutaneous fat depot and has attracted attention as a new independent risk factor for cardiovascular disease, dyslipidaemia, hypertension, arterial stiffness and metabolic syndrome [14–18]. However, few studies have examined the association between neck circumference and post-prandial hyperglycaemia. Those studies have shown that individuals with a neck circumference in the highest tertile have an increased risk of developing T2DM relative to those in the bottom tertile, even after adjustment for other measures of adiposity [19–21]. The results from our study support the role of neck circumference as an independent marker of post-prandial hyperglycaemia.

### Anthropometry identifies ß-cell dysfunction and impaired glucose curve shape

In addition to post-prandial hyperglycaemia patients in the highest tertile of any of the anthropometric measurements studied, had a significantly decreased insulin sensitivity and ß-cell function as compared with those in the lowest tertile. These findings highlight the association between central adiposity and ß-cell damage even at early stages of this pathological process. Along these lines, the 1-h glucose concentration was significantly higher in those subjects with the highest total and central adiposity and identified those with impaired beta-cell function, insulin resistance, and worse cardiovascular risk profile. Recent population-based studies have consistently shown that a 1-h post-OGTT glucose value over 8.6 mmol l^−1^ (155 mg dl^−1^) is a better predictor of incident T2DM, associated complications and mortality than fasting or 2-h levels [22–27]. These findings are of clinical importance as they suggest that the combination of 1-h post-prandial glucose and anthropometric measurements could be used as better predictive markers of IGT and T2DM compared to the full 2-h OGTT. Such an approach could not only reduce the number of patients undergoing OGTTs but also the number of glucose samples taken thus minimizing demands on resources.

## Limitations

As this study is cross-sectional, cause-effect relations among the degree of obesity assessed by anthropometric measurements and post-prandial hyperglycaemia and other cardiometabolic parameters cannot be established. The absence of prospective follow-up did not allow us to establish whether high values of anthropometric indices predicted the development of T2DM or the occurrence of major cardiovascular events. This was a single centre study so the results may not be extrapolated to other populations e.g. of different ethnic or socioeconomic backgrounds. Theoretically a potential selection bias could be present as the participants were not recruited randomly from the community but from patients attending a specialist clinic for nutritional interventions.

## Conclusions

In this large and comprehensively phenotyped cohort, one in four subjects had post-prandial hyperglycaemia despite normal fasting glycaemia. This highlights the importance of performing an OGTT as static normal fasting glycemia does not rule out post-prandial hyperglycaemia in this population. As an alternative to resource demanding OGTTs, there is an unmet need for readily available and inexpensive markers that can be used to screen patients that are most likely to have post-prandial hyperglycaemia and therefore benefit the most from an OGTT. In this study, anthropometric indices of central fat distribution were strongly and independently associated with an increased risk of post-prandial hyperglycaemia. These results support the association between central adiposity and the development of glucose derangements and demonstrate the clinical usefulness of anthropometric measurements as screening tools for the selection of patients who are most likely to benefit from an OGTT.

## Additional file

**Additional file 1: Table S1.** Adjusted mean 2-hours plasma glucose concentrations after a 75-g oral glucose tolerance test by categories of anthropometric measurement.

## Abbreviations

BMI: body mass index
DI: disposition index
HOMA: homeostasis model assessment
ISI: insulin sensitivity index or Matsuda index
OGTT: oral glucose tolerance test
QUICKI: quantitative insulin sensitivity check index
T2DM: type 2 diabetes mellitus

## References

[1] American Diabetes Association (2017). Classification and diagnosis of diabetes. Diabetes Care.

[2] Unwin N, Shaw J, Zimmet P, Alberti KG (2002). Impaired glucose tolerance and impaired fasting glycaemia: the current status on definition and intervention. Diabet Med.

[3] Bergman RN, Stefanovski D, Buchanan TA, Sumner AE, Reynolds JC, Sebring NG, Xiang AH, Watanabe RM (2011). A better index of body adiposity. Obesity (Silver Spring).

[4] Johansson G, Westerterp KR (2008). Assessment of the physical activity level with two questions: validation with doubly labeled water. Int J Obes (Lond).

[5] Trinder P (1969). Determination of blood glucose using an oxidase-peroxidase system with a non-carcinogenic chromogen. J Clin Pathol.

[6] Cersosimo E, Solis-Herrera C, Trautmann ME, Malloy J, Triplitt CL (2014). Assessment of pancreatic beta-cell function: review of methods and clinical applications. Curr Diabetes Rev.

[7] Blundell JE, Dulloo AG, Salvador J, Fruhbeck G (2014). Beyond BMI—phenotyping the obesities. Obes Facts.

[8] Gomez-Ambrosi J, Silva C, Catalan V, Rodriguez A, Galofre JC, Escalada J, Valenti V, Rotellar F, Romero S, Ramirez B (2012). Clinical usefulness of a new equation for estimating body fat. Diabetes Care.

[9] Gomez-Ambrosi J, Silva C, Galofre JC, Escalada J, Santos S, Millan D, Vila N, Ibanez P, Gil MJ, Valenti V (2012). Body mass index classification misses subjects with increased cardiometabolic risk factors related to elevated adiposity. Int J Obes (Lond).

[10] Walsh EI, Shaw J, Cherbuin N (2018). Trajectories of BMI change impact glucose and insulin metabolism. Nutr Metab Cardiovasc Dis.

[11] Gomez-Ambrosi J, Silva C, Galofre JC, Escalada J, Santos S, Gil MJ, Valenti V, Rotellar F, Ramirez B, Salvador J, Fruhbeck G (2011). Body adiposity and type 2 diabetes: increased risk with a high body fat percentage even having a normal BMI. Obesity (Silver Spring).

[12] Bennasar-Veny M, Lopez-Gonzalez AA, Tauler P, Cespedes ML, Vicente-Herrero T, Yanez A, Tomas-Salva M, Aguilo A (2013). Body adiposity index and cardiovascular health risk factors in Caucasians: a comparison with the body mass index and others. PLoS ONE.

[13] Rodriguez A, Ezquerro S, Mendez-Gimenez L, Becerril S, Fruhbeck G (2015). Revisiting the adipocyte: a model for integration of cytokine signaling in the regulation of energy metabolism. Am J Physiol Endocrinol Metab.

[14] Vallianou NG, Evangelopoulos AA, Bountziouka V, Vogiatzakis ED, Bonou MS, Barbetseas J, Avgerinos PC, Panagiotakos DB (2013). Neck circumference is correlated with triglycerides and inversely related with HDL cholesterol beyond BMI and waist circumference. Diabetes Metab Res Rev.

[15] Fantin F, Comellato G, Rossi AP, Grison E, Zoico E, Mazzali G, Zamboni M (2017). Relationship between neck circumference, insulin resistance and arterial stiffness in overweight and obese subjects. Eur J Prev Cardiol.

[16] Assyov Y, Gateva A, Tsakova A, Kamenov Z (2017). A comparison of the clinical usefulness of neck circumference and waist circumference in individuals with severe obesity. Endocr Res.

[17] Lee JJ, Pedley A, Therkelsen KE, Hoffmann U, Massaro JM, Levy D, Long MT (2017). Upper body subcutaneous fat is associated with cardiometabolic risk factors. Am J Med.

[18] Zhou JY, Ge H, Zhu MF, Wang LJ, Chen L, Tan YZ, Chen YM, Zhu HL (2013). Neck circumference as an independent predictive contributor to cardio-metabolic syndrome. Cardiovasc Diabetol.

[19] Freedman DS, Rimm AA (1989). The relation of body fat distribution, as assessed by six girth measurements, to diabetes mellitus in women. Am J Public Health.

[20] Khalangot M, Gurianov V, Okhrimenko N, Luzanchuk I, Kravchenko V (2016). Neck circumference as a risk factor of screen-detected diabetes mellitus: community-based study. Diabetol Metab Syndr.

[21] Cho NH, Oh TJ, Kim KM, Choi SH, Lee JH, Park KS, Jang HC, Kim JY, Lee HK, Lim S (2015). Neck circumference and incidence of diabetes mellitus over 10 years in the Korean Genome and Epidemiology Study (KoGES). Sci Rep.

[22] Jagannathan R, Sevick MA, Li H, Fink D, Dankner R, Chetrit A, Roth J, Bergman M (2016). Elevated 1-hour plasma glucose levels are associated with dysglycemia, impaired beta-cell function, and insulin sensitivity: a pilot study from a real world health care setting. Endocrine.

[23] Abdul-Ghani MA, Lyssenko V, Tuomi T, DeFronzo RA, Groop L (2009). Fasting versus postload plasma glucose concentration and the risk for future type 2 diabetes: results from the Botnia Study. Diabetes Care.

[24] Alyass A, Almgren P, Akerlund M, Dushoff J, Isomaa B, Nilsson P, Tuomi T, Lyssenko V, Groop L, Meyre D (2015). Modelling of OGTT curve identifies 1 h plasma glucose level as a strong predictor of incident type 2 diabetes: results from two prospective cohorts. Diabetologia.

[25] Pareek M, Bhatt DL, Nielsen ML, Jagannathan R, Eriksson KF, Nilsson PM, Bergman M, Olsen MH (2017). Enhanced predictive capability of a 1-hour oral glucose tolerance test: a prospective population-based cohort study. Diabetes Care.

[26] Bianchi C, Miccoli R, Trombetta M, Giorgino F, Frontoni S, Faloia E, Marchesini G, Dolci MA, Cavalot F, Cavallo G (2013). Elevated 1-hour postload plasma glucose levels identify subjects with normal glucose tolerance but impaired beta-cell function, insulin resistance, and worse cardiovascular risk profile: the GENFIEV study. J Clin Endocrinol Metab.

[27] Fiorentino TV, Marini MA, Andreozzi F, Arturi F, Succurro E, Perticone M, Sciacqua A, Hribal ML, Perticone F, Sesti G (2015). One-hour postload hyperglycemia is a stronger predictor of type 2 diabetes than impaired fasting glucose. J Clin Endocrinol Metab.

