# Supplementary material for: Discriminatory ability of anthropometric measurements of central fat distribution for prediction of post-prandial hyperglycaemia in patients with normal fasting glucose: the DICAMANO Study
Source: J Transl Med. 2019 Feb 18;17:48. doi: 10.1186/s12967-019-1787-5 (PMC6379947; doi:10.1186/s12967-019-1787-5)
Supplement: Supplementary file 1 — Additional file 1: Table S1. Adjusted mean 2-hours plasma glucose concentrations after a 75-g oral glucose tolerance test by categories of anthropometric measurement. [file 12967_2019_1787_MOESM1_ESM.docx]

**Table S1.** Adjusted mean 2-hours plasma glucose concentrations after a 75-g oral glucose tolerance test by categories of anthropometric measurement.

| **Anthropometric measurement** | **Category** | **Adjusted mean 30 min glucose (95% CI)*** | **P value (ANCOVA)** | **Adjusted mean 1-hour glucose (95% CI)*** | **P value (ANCOVA)** | **Adjusted mean 2-hours glucose (95% CI)*** | **P value (ANCOVA)** |
| --- | --- | --- | --- | --- | --- | --- | --- |
| **Body mass index (kg/m^2^)** | Low (25.1-29.9) | 7.8 (7.6, 8.0) | 0.52 | 7.7 (7.4, 8.1) | **<0.001** | 6.2 (5.8, 6.5) | **<0.001** |
|  | Intermediate (30.0-34.9) | 7.7 (7.6, 7.9) |  | 8.3 (7.9, 8.6) |  | 6.9 (6.7, 7.2) |  |
|  | High (>35) | 7.9 (7.7, 8.0) |  | 8.8 (8.5, 9.1) |  | 7.2 (6.9, 7.5) |  |
| **Body adiposity index** | Low (21.3 – 33.3) | 7.8 (7.6, 7.9) | 0.553 | 8.4 (8.1, 8.7) | 0.01 | 6.9 (6.6, 7.2) | **0.005** |
|  | Intermediate (33.4 – 38.5) | 7.8 (7.6, 8.0) |  | 7.9 (7.6, 8.3) |  | 6.4 (6.2, 6.2) |  |
|  | High (38.7 - 74) | 7.9 (7.7, 8.1) |  | 8.7 (8.3, 9.0) |  | 7.2 (6.9, 7.5) |  |
| **Neck circumference** | Low (30-35) | 7.7 (7.5, 7.8) | 0.130 | 7.6 (7.3, 7.9) | **<0.001** | 6.2 (5.9, 6.6) | **<0.001** |
|  | Intermediate (36-40) | 7.9 (7.8, 8.1) |  | 8.6 (8.2, 8.8) |  | 6.8 (6.7, 7.3) |  |
|  | High (41-57) | 7.8 (7.6, 8.0) |  | 8.9 (8.6, 9.3) |  | 7.3 (7.0, 7.7) |  |
| **Waist circumference (cm)** | Low (73-100) | 7.8 (7.6, 7.9) | 0.806 | 7.6 (7.3, 7.9) | **<0.001** | 6.2 (5.9, 6.5) | **<0.001** |
|  | Intermediate (101-114) | 7.8 (7.7, 8.0) |  | 8.5 (8.2, 8.8) |  | 7.1 (6.8, 7.4) |  |
|  | High (115-170) | 7.8 (7.7, 8.0) |  | 9.0 (8.7, 9.3) |  | 7.3 (7.0, 7.6) |  |
| **Waist-to-hip ratio** | Low (0.71-0.88) | 7.7 (7.5, 7.9) | 0.238 | 7.7 (7.4, 8.0) | **<0.001** | 6.4 (6.2, 6.8) | **<0.001** |
|  | Intermediate (0.89-0.97) | 7.8 (7.7, 8.0) |  | 8.3 (8.0, 8.6) |  | 6.6 (6.3, 6.8) |  |
|  | High (0.98-1.30) | 7.9 (7.7, 8.1) |  | 9.1 (8.8, 9.4) |  | 7.6 (7.3, 7.9) |  |
| **Waist-to-height ratio** | Low (0.47-0.61) | 7.7 (7.5, 7.8) | 0.08 | 7.7 (7.4, 7.9) | **<0.001** | 6.5 (5.9, 6.4) | **<0.001** |
|  | Intermediate (0.62-0.68) | 7.8 (7.7, 8.1) |  | 8.4 (8.1, 8.7) |  | 6.9 (6.7, 7.3) |  |
|  | High (0.69-1.11) | 7.9 (7.8, 8.1) |  | 9.2 (8.8, 9.5) |  | 7.5 (7.2, 7.8) |  |

*Adjusted for fasting glucose concentrations. ANCOVA, analysis of covariance. CI, confidence interval. OGTT, oral glucose tolerance test. .
